# The Epstein-Barr virus encoded LMP1 oncoprotein modulates cell adhesion via regulation of activin A/TGFβ and β1 integrin signalling

**DOI:** 10.1038/srep19533

**Published:** 2016-01-19

**Authors:** Mhairi A. Morris, Christopher W. Dawson, Louise Laverick, Alexandra M. Davis, Joe P. R. Dudman, Sathuwarman Raveenthiraraj, Zeeshan Ahmad, Lee-Fah Yap, Lawrence S. Young

**Affiliations:** 1 Faculty of Health and Life Sciences, Hawthorn Building, De Montfort University, The Gateway, Leicester, LE1 9BH; 2 Institute for Cancer Studies, School of Cancer Sciences, The University of Birmingham, Vincent Drive, Edgbaston, Birmingham, B15 2TT; 3 Department of Medicine, University of Melbourne, Clinical Sciences, Royal Melbourne Hospital, Royal Parade, Parkville, Victoria 3050; 4 Department of Oral Biology & Biomedical Sciences and Oral Cancer Research & Coordinating Centre, Faculty of Dentistry, University of Malaya, 50603, Kuala Lumpur, Malaysia; 5 Warwick Medical School, University of Warwick, Coventry, CV4 8UW.

## Abstract

Approximately 20% of global cancer incidence is causally linked to an infectious agent. Epstein-Barr virus (EBV) accounts for around 1% of all virus-associated cancers and is associated with nasopharyngeal carcinoma (NPC). Latent membrane protein 1 (LMP1), the major oncoprotein encoded by EBV, behaves as a constitutively active tumour necrosis factor (TNF) receptor activating a variety of signalling pathways, including the three classic MAPKs (ERK-MAPK, p38 MAPK and JNK/SAPK). The present study identifies novel signalling properties for this integral membrane protein via the induction and secretion of activin A and TGFβ1, which are both required for LMP1’s ability to induce the expression of the extracellular matrix protein, fibronectin. However, it is evident that LMP1 is unable to activate the classic Smad-dependent TGFβ signalling pathway, but rather elicits its effects through the non-Smad arm of TGFβ signalling. In addition, there is a requirement for JNK/SAPK signalling in LMP1-mediated fibronectin induction. LMP1 also induces the expression and activation of the major fibronectin receptor, α5β1 integrin, an effect that is accompanied by increased focal adhesion formation and turnover. Taken together, these findings support the putative role for LMP1 in the pathogenesis of NPC by contributing to the metastatic potential of epithelial cells.

EBV is a ubiquitous human gammaherpesvirus that infects approximately 95% of the population worldwide, persisting as a lifelong, largely asymptomatic infection. However, aberrant latent infection with EBV is linked to the pathogenesis of various lymphoid and epithelial malignancies, including endemic Burkitt’s lymphoma, Hodgkin’s lymphoma, NPC and a proportion of EBV-positive gastric carcinomas^1^. Unlike the differentiated form of NPC, the non-keratinising and undifferentiated forms of NPC are unique among squamous cell carcinomas of the head and neck due to their universal association with EBV infection^2^. NPC is endemic to areas of China and South-East Asia, with a peak incidence of 20–30 cases per 100,000 per annum, while intermediate incidences are observed in North Africa and the Mediterranean basin^3^. While the contribution of EBV infection to the pathogenesis of NPC is still unclear, a number of EBV latent genes with proven growth modulatory potential are expressed within tumour cells. Here, EBV latent gene expression is restricted to EBV-nuclear antigen 1 (EBNA1), the non-coding EBER1 and EBER2 RNAs, the BART family of microRNAs, and variable expression of the latent membrane proteins, LMP1, LMP2A and LMP2B^4^. Although limited, analysis of rare premalignant lesions of the nasopharynx from patients in high-risk NPC regions has revealed the presence of monoclonal EBV genomes and detectable levels of LMP1 expression, suggesting a role for this viral oncogene in the early stages of NPC pathogenesis^5^.

LMP1 is a 66 kDa integral membrane protein that shares signalling properties with members of the TNF receptor superfamily. LMP1 has been shown to engage the three classic mitogen-activated protein kinases (MAPKs): ERK-MAPK, p38 MAPK and JNK/SAPK, the canonical and non-canonical NF-κB pathways, and the PI3K pathway^6^. LMP1 behaves as a classical oncogene, transforming rodent fibroblasts *in vitro* and rendering them tumourigenic *in vivo*^4^,^7^. In epithelial cells, LMP1 exerts similar growth-enhancing effects, promoting cell growth, stimulating migration and invasion, and modulating squamous epithelial differentiation^8^,^9^. Moreover, LMP1 is known to induce the expression of matrix metalloproteinases *in vitro*, which are secreted by tumour cells to breakdown the surrounding tissue stroma thereby enhancing tumour cell invasive potential^10^,^11^. In Madin-Darby Canine Kidney (MDCK) epithelial cells, LMP1 can profoundly alter their morphology, inducing a phenomenon known as epithelial-to-mesenchymal transition (EMT)^12^, a phenomenon that may be linked to the ability of LMP1 to induce cancer stem/progenitor cell-like properties in certain epithelial cell lines^13^. When targeted to the epidermis of transgenic mice, LMP1 was found to induce a form of epidermal hyperplasia, similar to that observed in psoriasis^14^,^15^, findings which are supported by the observation that LMP1 induces an inflammatory keratinocyte wounding response in epithelial cells *in vitro*^10^. LMP1 is also known to induce various proteins with pro-angiogenic functions, such as MMP1 and VEGF^16^,^17^, and VEGF-C has been shown to induce lymphangiogenesis in NPC; however, the authors do not see a correlation between LMP1 expression and VEGF-C expression^18^. Collectively, these findings support a role for LMP1 in the pathogenesis of NPC by modulating the cellular phenotype.

The TGFβ superfamily comprises a range of pleiotropic growth factors produced by a diverse range of cell types that modulate cell migration, cell adhesion, proliferation, differentiation and survival^19^. TGFβ1 is the prototypic member but others include TGFβ2 and TGFβ3, and the activins. Activin A and TGFβ primarily signal through very similar pathways involving the regulatory signalling molecules called Smads. Depending on the different transcription factors they interact with, Smads can either stimulate or repress the transcription of their target genes^19^,^20^. Alternative, Smad-independent signalling pathways also exist for both activin A and TGFβ. For instance, activin A can signal through the RhoA-ROCK-MEKK1-JNK and MEKK1-p38 signalling cascades, which are implicated in the actin cytoskeleton remodelling and cell migration involved in epithelial morphogenesis^21^; whilst the small GTPase, Ras, and the MAPKs, ERK, p38 and JNK, are all important downstream factors in Smad-independent TGFβ signalling^22^.

Both activin A and TGFβ are implicated in the processes of wound healing and tumourigenesis^23^,^24^. Indeed, the expression of various activin family members has been detected in a number of cancers, for example, prostate cancer^25^, breast cancer^26^, and retinoblastoma^27^. Both molecules have been shown to stimulate the expression and secretion of ECM proteins such as fibronectin and type I collagen^23^,^28^. The integrins are a family of cell surface receptors that bind to various ECM proteins: in cell culture, or in response to wounding, fibronectin specifically binds α5β1^29^.

In the current study, the induction of activin A and TGFβ by LMP1 was investigated further, with particular emphasis on their role in generating a fibrotic response. Microarray analysis of tetracycline-inducible SCC12F cells expressing LMP1 identified TGFβ and activin A as LMP1 target genes in keratinocytes^10^. However, LMP1 has previously been shown to inhibit the activation of TGFβ/Smad signalling in epithelial cells and fibroblasts, via an NF-κB-dependent mechanism^30^,^31^. The apparent contradiction between these studies supports the requirement for further investigation into the effects of LMP1-induced activin A and/or TGFβ on epithelial cells *in vitro*.

The present study demonstrates that LMP1-mediated induction of activin A and/or TGFβ are required to induce the expression and secretion of fibronectin, a phenotype associated with the fibrotic response in wounded keratinocytes in preparation for migration and invasion, and a precursor to neoplasia. However, contrary to expectations, the findings presented here demonstrate that Smad-dependent transcription is inhibited in LMP1-expressing cells, and furthermore confirm the Smad-independency of fibronectin induction by LMP1. Furthermore, this study demonstrates that LMP1-mediated fibronectin induction is abrogated by inhibiting JNK/SAPK signalling, corroborating previous findings. In addition, an increase in the activation of β1 integrins and the expression of α5 integrins on the surface of LMP1-expressing cells is observed. Since α5β1 integrin is the major fibronectin receptor, these findings therefore support the hypothesis that LMP1 may be upregulating the expression and secretion of fibronectin and cell surface expression of its receptor in order to facilitate invasion and metastasis. Finally, the findings highlight a role for activin A and/or TGFβ signalling in LMP1-enhanced cell adhesion and spreading. Taken together, these data support a role for LMP1 in mediating an invasive and metastatic phenotype in epithelial cells *in vitro*, thereby suggesting a putative role for LMP1 in the pathogenesis of NPC *in vivo*. This may have wider implications for the identification of therapeutic targets for NPC and other EBV-associated cancers, which bear many common features.

## Results

### LMP1 increases the expression and secretion of activin A and TGFβ and enhances fibronectin synthesis, secretion and expression

**Figure 1:**
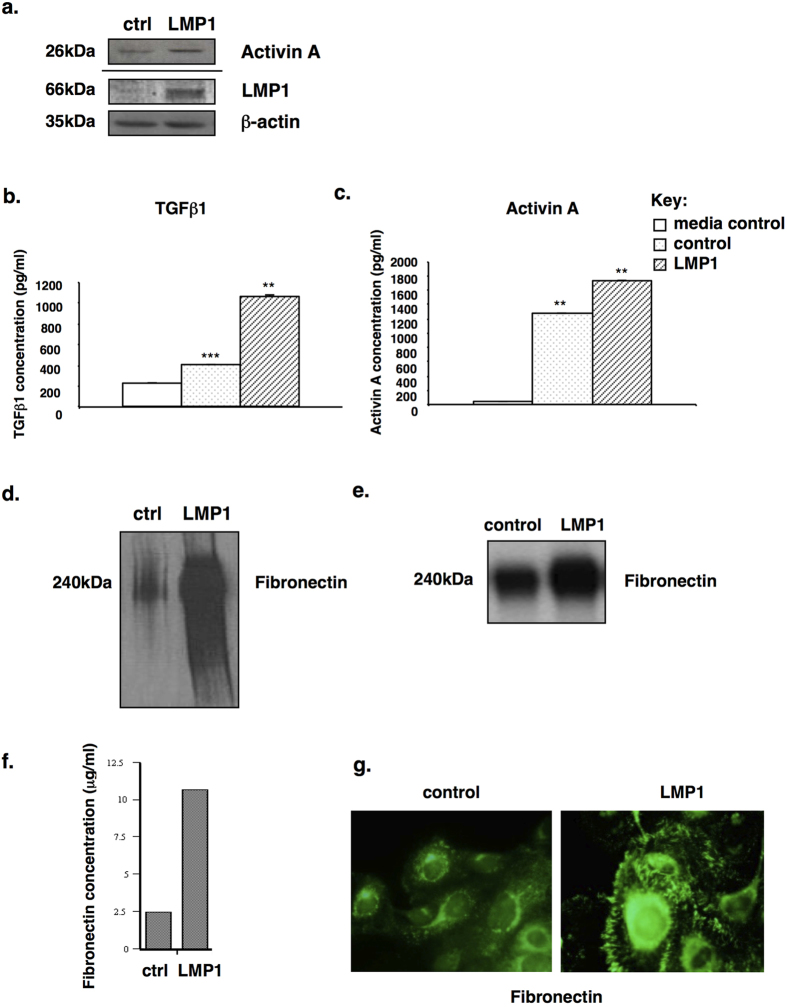
Analysis of expression of activin A (**a**) and secretion of TGFβ1 (**b**) and activin A (**c**) in LMP1-expressing SCC12F cells compared with control cells. The student’s t-test confirmed the statistical significance of the difference in cytokine secretion relative to the control, with p values of <0.01 and <0.001, denoted by two and three asterisks, respectively. LMP1 expression in SCC12F cells is associated with increased fibronectin synthesis, secretion and deposition into cell-associated matrix, as measured by (**d**) Western blotting, (**e**) ^35^S Methionine labelling and immunoprecipitation, (**f**) ELISA, and (**g**) immunofluorescence staining.

Building on previous observations outlining the ability of LMP1 to induce the expression of activin A and TGFβ in a tet-LMP1 inducible system^10^, the expression and secretion of activin A and TGFβ was investigated in SCC12F epithelial cells stably expressing LMP1. Immunoblotting confirmed the upregulation of activin A at the protein level in LMP1-expressing SCC12F cells (Fig. 1a). Furthermore, ELISA confirmed a 2.61-fold increase in TGFβ1 levels and a 1.37-fold increase in activin A levels secreted in conditioned medium taken from LMP1-expressing cells (Fig. 1b,c; with p-values <0.01 and <0.001, denoted by two and three asterisks, respectively). These results are in agreement with previously published data that the levels of activin A and TGFβ1 were up-regulated in micro-dissected primary NPC cells compared with normal epithelium^32^.

As shown in Fig. 1d, Western blotting confirmed that LMP1-expressing SCC12F cells expressed significantly higher levels of cellular fibronectin compared to control cells. Furthermore, ^35^S-Methionine labelling and immunoprecipitation confirmed increased translation of fibronectin in LMP1-expressing cells (Fig. 1e), while ELISA (Fig. 1f) confirmed that LMP1-expressing cells synthesised and secreted higher levels of fibronectin compared to control cells. Interestingly, immunofluorescence staining of cells cultured *in situ* confirmed that LMP1-expressing cells deposited higher amounts of fibronectin into their extracellular matrix than control cells (Fig. 1g) suggesting that LMP1 modulates ECM protein incorporation into cell-associated matrix. Given that TGFβ and activin A are known to participate in fibrotic responses under conditions of chronic inflammation, and that LMP1 can upregulate the expression of activin A and TGFβ, it is logical to hypothesise that LMP1-mediated fibronectin induction may be elicited by activin A and/or TGFβ.

### LMP1-mediated fibronectin induction is dependent on activin A and/or TGFβ

Both activin A and TGFβ are known to stimulate the expression and secretion of ECM proteins, including fibronectin^23^,^33^. Moreover, both cytokines have been linked to fibrosis of the liver, lungs and kidneys^34^,^35^. According to a study published in 2002 by Laping and colleagues, the TGFβ-mediated induction of fibronectin mRNA expression is not significant until 16 hours post treatment; thus, this time-point was used in the current study^36^. Furthermore, TGFβ-mediated fibronectin induction has been shown to be independent of Smad4 and instead requires a signal from JNK/SAPK, but not ERK-MAPK or p38 MAPK, in human fibrosarcoma cell lines^33^. In order to assess the contribution of LMP1-induced activin A and/or TGFβ to the induction of fibronectin expression, control and LMP1-expressing cells were treated with the small molecule inhibitor of the activin A and TGFβ type I receptor, SB431542^37^.

**Figure 2:**
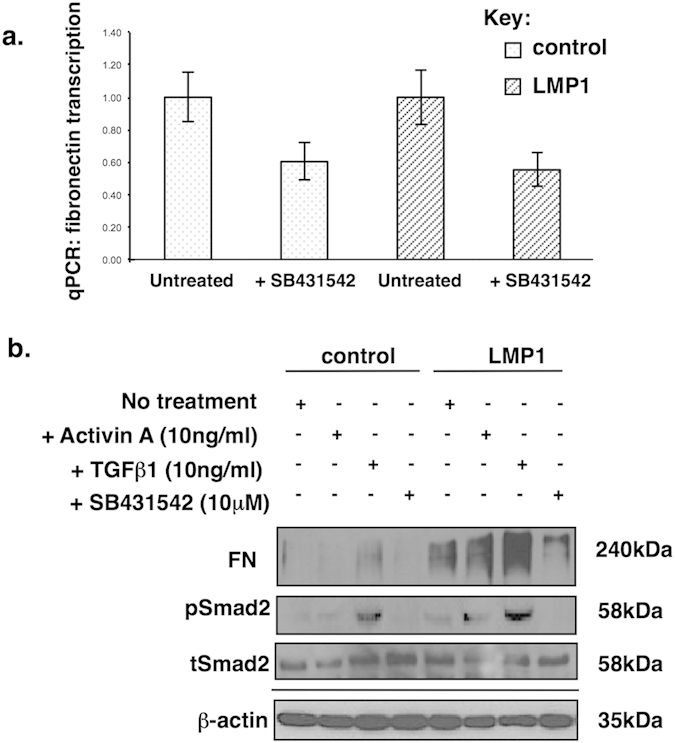
Treatment of control and LMP1-expressing SCC12F cells with the activin A/TGFβ receptor (ALK5) inhibitor, SB431542, reduces fibronectin transcription as measured by qPCR (**a**); and reduces fibronectin protein expression (**b**). Western blotting confirmed abrogation of Smad2 phosphorylation by addition of SB431542 with no effect on total Smad2 levels.

Figure 2 demonstrates the requirement for signalling through activin A and/or TGFβ1 for the induction of fibronectin in LMP1-expressing cells at the mRNA level (Fig. 2a). Moreover, the addition of exogenous activin A and TGFβ1 augments LMP1’s ability to induce fibronectin expression at the protein level (Fig. 2b). A role for activin A or TGFβ in this response was confirmed, as fibronectin expression was abrogated by addition of the activin A/TGFβ inhibitor, SB431542.

### LMP1 inhibits Smad-dependent transcription and LMP1-mediated fibronectin induction occurs via a Smad-independent mechanism

**Figure 3:**
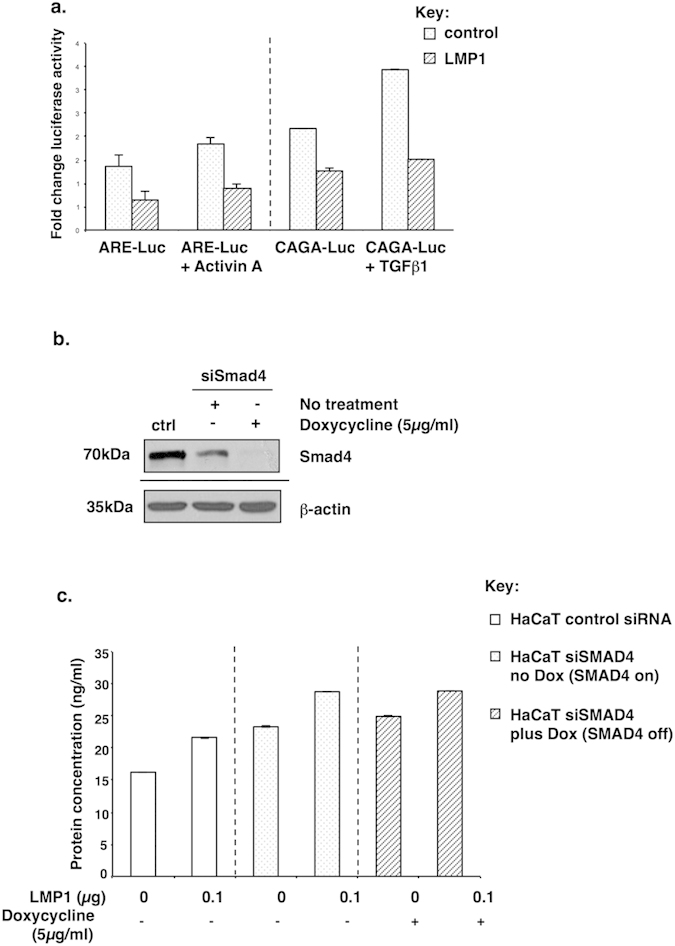
Renilla luciferase reporter assays confirmed LMP1’s inability to activate Smad2/4-mediated gene transcription (ARE-Luc) and Smad3/4-mediated gene transcription (CAGA-Luc; (**a**). Effectiveness of Smad4 siRNA upon addition of 5 g/ml doxycycline was confirmed by Western blotting (**b**). The ability of LMP1 to induce fibronectin secretion was confirmed by ELISA to be Smad-independent in HaCaT cell lines stably expressing tetracycline-inducible siRNA to Smad4 (**c**).

It has previously been reported that LMP1 inhibits TGFβ-mediated growth arrest and the activation of Smad-dependent signalling in epithelial cells and fibroblasts via an NF-κB-dependent mechanism^30^,^31^; however, as yet there is no published data concerning the phosphorylation status of the downstream signalling intermediates, the Smads, in response to LMP1 expression. Indeed, in agreement with published observations, data presented here shows that LMP1 failed to activate Smad-dependent transcription in SCC12F cells, as assessed by dual luciferase reporter assays using either a Smad2/3-dependent activin response element (ARE-Luc) which binds Smad2 and Smad3^38^ or the Smad3/4-binding sequences, termed CAGA boxes (CAGA-Luc) which binds Smad3 and Smad4^39^ (Fig. 3a). It is interesting to note, however, that despite this apparent inhibition of Smad-dependent transcription, LMP1 expression still enhanced basal Smad2 phosphorylation – a general measure of activin A and TGFβ activity (Fig. 2b).

As a consequence of this observation, the requirement for Smad activity in LMP1-mediated fibronectin induction was also assessed. Indeed, fibronectin induction has been reported to be a Smad4-independent TGFβ target gene^33^. Since Smad4 expression is absolutely required to transduce Smad-dependent signalling from both activin and TGFβ receptors^40^, the Smad-independency of LMP1-mediated induction of fibronectin was investigated using HaCaT cells stably expressing a tetracycline-regulatable Smad4 siRNA (Fig. 3b). The cells were then transiently transfected with low levels of LMP1 to be more representative of NPC, following which the ability of LMP1 to enhance fibronectin secretion in the presence and absence of Smad4 was assessed. As demonstrated in Fig. 3c, LMP1’s ability to enhance fibronectin secretion did not appear to be affected by Smad4 silencing upon addition of doxycycline.

### LMP1-mediated fibronectin induction is promoted via the JNK/SAPK pathway

Since LMP1-mediated fibronectin induction requires a signal from activin A and/or TGFβ, yet Smad-dependent transcription is inhibited by LMP1, the relative contribution of various Smad-independent pathways on this cellular phenotype was subsequently investigated. In addition to the canonical Smad-dependent pathway, both activin A and TGFβ have been shown to signal via Smad-independent, yet receptor-dependent, pathways including the three MAPK pathways, JNK/SAPK, ERK-MAPK and p38 MAPK, and also through the PI3K/Akt signalling pathway^41^.

**Figure 4:**
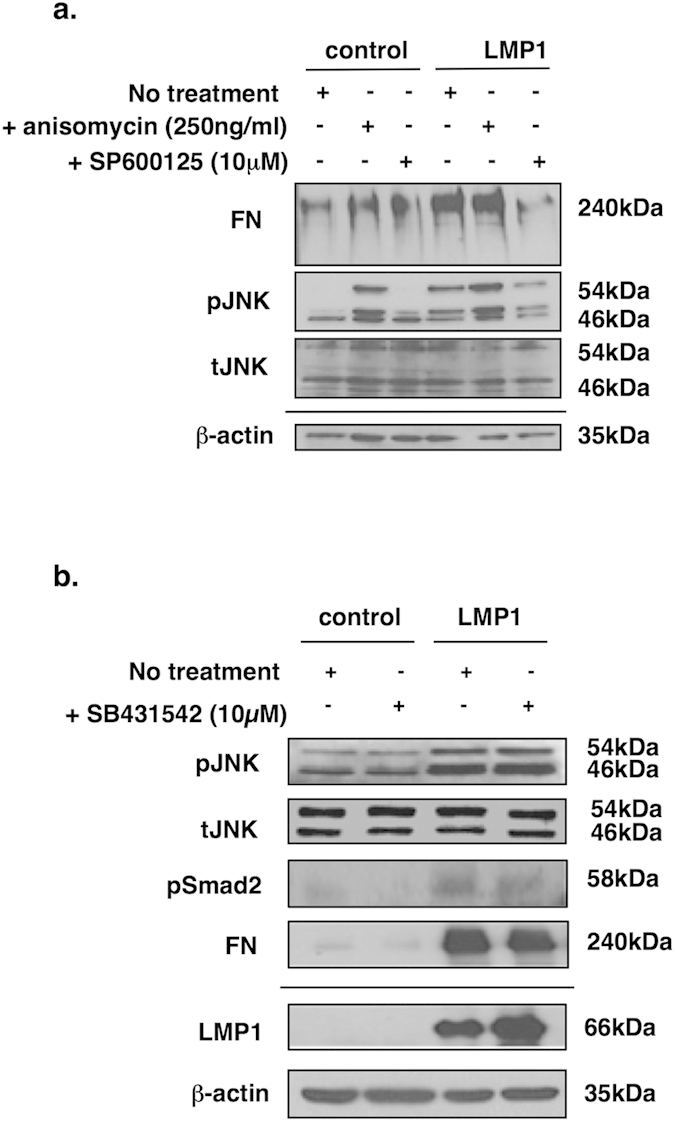
Western blotting confirmed the requirement for LMP1-mediated JNK/SAPK signalling in the induction of fibronectin using anisomycin, which stimulates JNK/SAPK signalling, and SP600125, a specific small molecule inhibitor of the JNK/SAPK pathway (a). The activation of JNK/SAPK signalling by LMP1 is not affected by addition of the activin A/TGFβ inhibitor, SB431542 (**b**).

Using a panel of small molecule inhibitors, the common signalling pathways engaged by LMP1 that may be responsible for LMP1-mediated fibronectin induction were interrogated. The results revealed a role for JNK/SAPK signalling in LMP1’s ability to induce fibronectin expression (Fig. 4a). Moreover, it is evident that inhibition of activin A and/or TGFβ does not affect LMP1’s ability to activate JNK/SAPK signalling (Fig. 4b).

### LMP1 activates and upregulates the cell surface expression of the fibronectin receptor, integrin α5β1

**Figure 5:**
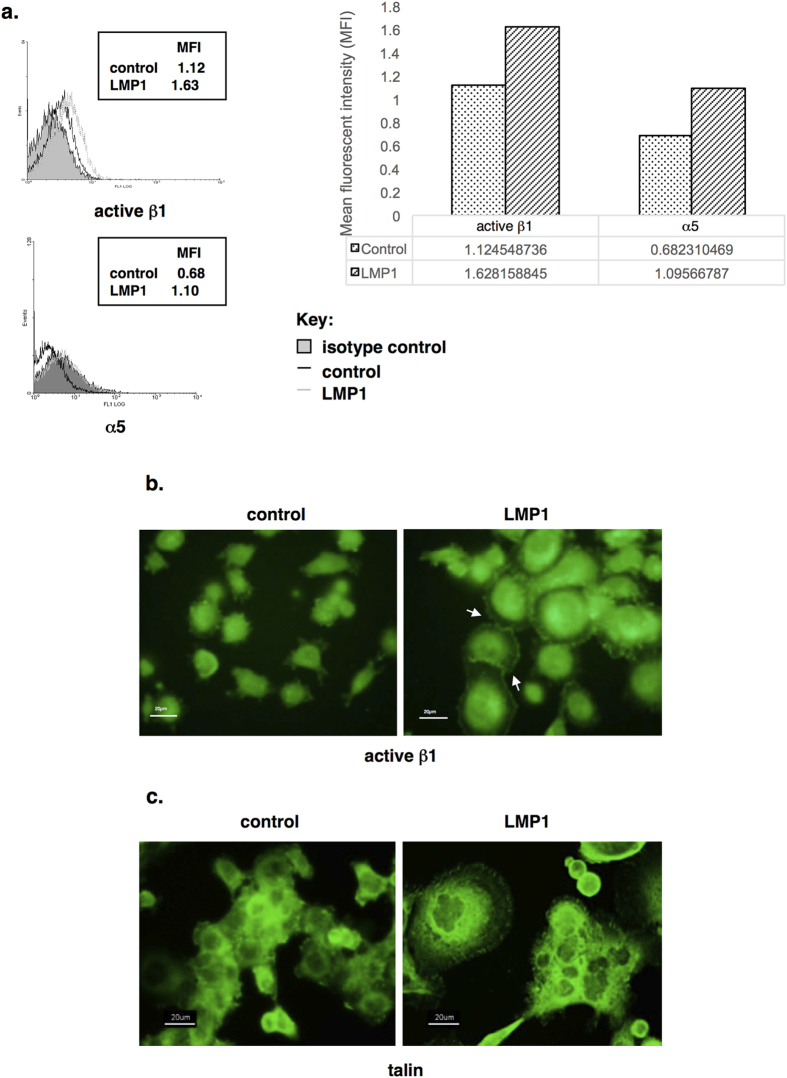
Flow cytometry reveals increased levels of active β1 integrins and integrin α5 on the surface of LMP1-expressing SCC12Fs when compared with controls. Calculation of the Mean Fluorescent Intensity (MFI) revealed a 1.45-fold increase in active integrin β1, and a 1.61-fold increase in integrin α5 (**a**). Immunofluorescence staining reveals elevated, punctate staining of active β1 integrins (**b**) and talin (**c**) at sites of focal adhesion in LMP1-expressing cells.

One of the possible downstream consequences of LMP1’s ability to induce fibronectin could be to activate the cognate receptors, leading to a host of other signalling events, for example to enhance cell survival and promote migration. Indeed, LMP1 expression results in an increase in the number of active β1 integrins found on the surface of SCC12F cells, as assessed by flow cytometry using an antibody that recognises conformationally “active” β1 integrins (Fig. 5a). Moreover, the cell surface levels of the α5 integrin subunit were also elevated in response to LMP1 expression (Fig. 5a). The enhanced activation of β1 integrins was confirmed by immunofluorescence staining of cells grown *in situ*, which demonstrated an increased levels of conformationally “active” β1 integrins on the surface of LMP1-expressing cells, particularly at sites of focal adhesion (Fig. 5b; white arrows). This is supported by the finding that the expression of talin, an associated protein that is only recruited to sites of integrin activation^42^, is also enhanced in LMP1-expressing cells where it can be seen as punctate staining around the edge of the adherent cells, which are far larger than the control counterparts, indicative of enhanced adhesion (Fig. 5c). These data support the hypothesis that LMP1 may increase the synthesis and secretion of fibronectin, whilst also upregulating the cell surface expression of α5β1 integrins, to facilitate its own autocrine pro-survival and pro-metastatic signalling.

### LMP1 promotes cell attachment and spreading and increases focal adhesion formation in response to adhesion to fibronectin

**Figure 6:**
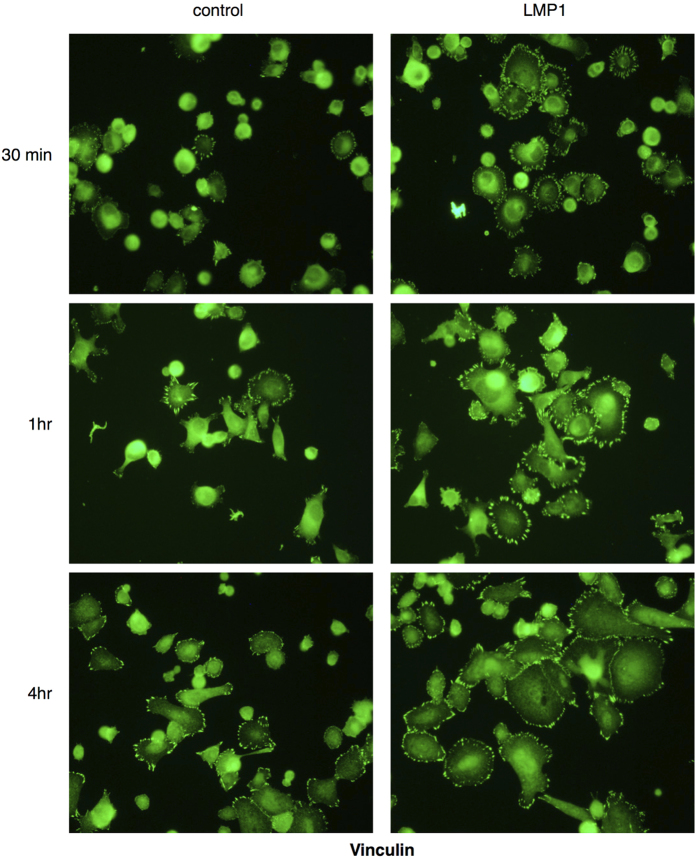
Immunofluorescence staining for vinculin revealed increased rates of attachment of LMP1-expressing SCC12F cells to fibronectin and increased focal adhesion formation at the edge of spreading cells.

Formation and disassembly of focal adhesions, also known as focal adhesion turnover, is a dynamic process that occurs during cell migration and is mediated via integrin receptor engagement with ECM proteins. To confirm that LMP1 stimulates the expression and activity of β1 integrins, control and LMP1-expressing cells were serum starved overnight, recovered as single cell suspensions and plated onto fibronectin-coated slides. At various times, cells were fixed and stained for vinculin, a component of focal adhesions. As shown in Fig. 6, LMP1-expressing cells adhered more rapidly to fibronectin than control cells, assembling robust focal adhesions, as marked by vinculin staining. At later time points (1hr–4hrs), LMP1-expressing cells attained higher levels of spreading. These findings confirmed that LMP1 stimulates integrin activity, which facilitates cell attachment and spreading.

### LMP1 activates ERK-MAPK at the leading edge of invadopodia in response to adhesion onto fibronectin

**Figure 7:**
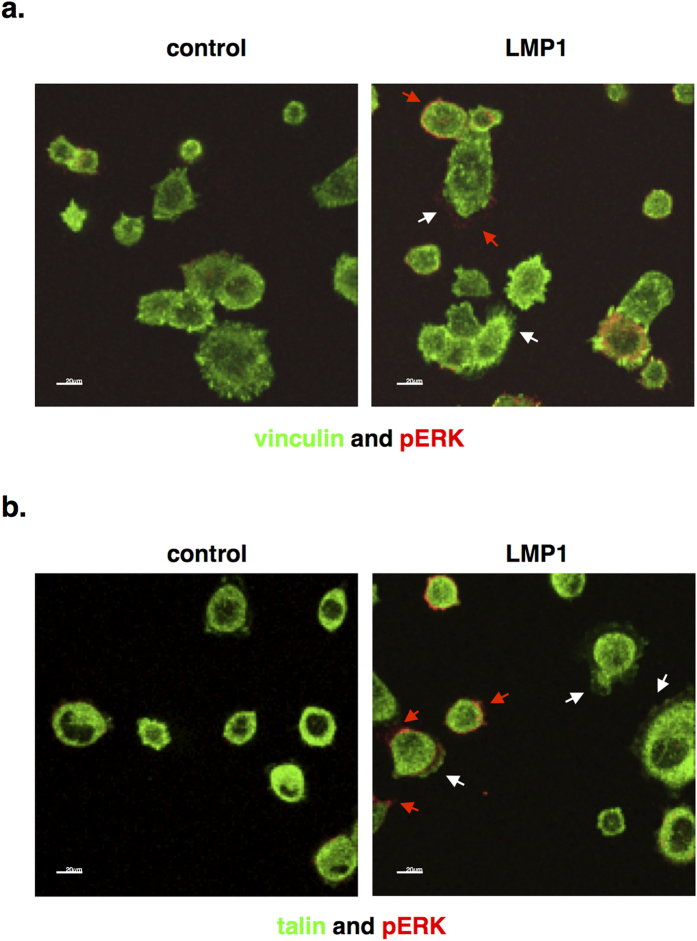
Immunofluorescence staining and confocal microscopy reveal increased levels of active ERK-MAPK (red arrows), at the edge of spreading cells, alongside vinculin (a) and talin (**b**; both white arrows) in LMP1-expressing cells when plated onto fibronectin-coated slides.

ERK-MAPK is known to be activated at points of focal adhesion turnover and serves as a marker for increased integrin-mediated adhesion^43^. Further to our previous observations that LMP1 can enhance the rate of cell motility in an ERK-dependent manner^44^, ERK-MAPK activation in response to LMP1-mediated adhesion to fibronectin was investigated. Figure 7a shows distinctly elevated levels of active, phosphorylated ERK-MAPK at the edge of the cell protrusions in LMP1-expressing cells (red arrows). Interestingly, the activated ERK-MAPK does not always co-localise with vinculin (white arrows) – a component of the focal adhesion complex – a phenomenon that is likely due to the timing of focal adhesion complex formation and turnover. Also, Fig. 7b demonstrates some degree of co-localisation between talin (white arrows) and phosphorylated ERK-MAPK (red arrows).

### The increased rate of cell attachment and spreading onto fibronectin-coated slides observed with LMP1 expression can be abrogated by inhibiting activin A/TGFβ signalling

**Figure 8:**
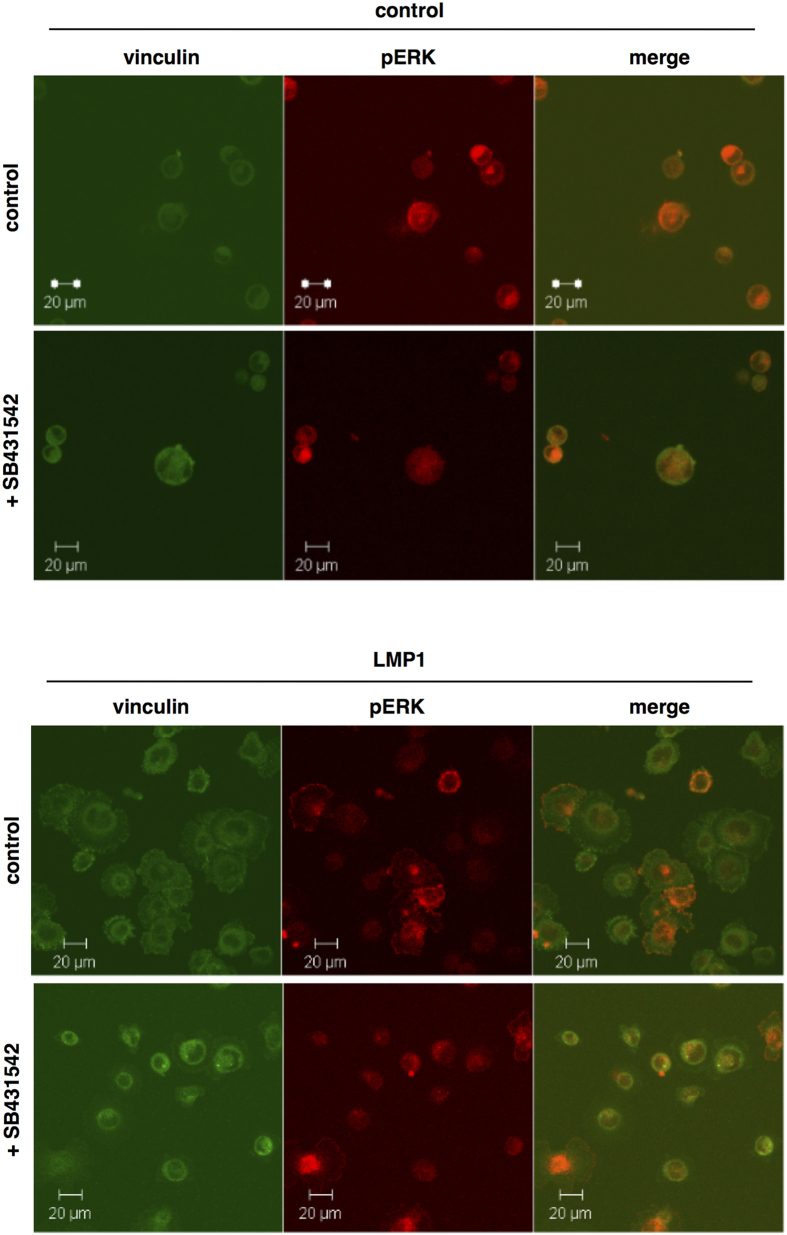
The enhanced rate of adhesion and spreading onto fibronectin-coated slides observed in LMP1-expressing SCC12Fs is abrogated by addition of the activin A/TGFβ small molecule inhibitor, SB431542, as visualised by immunofluorescence staining for active ERK-MAPK (red) and vinculin (green).

Further to these observations, data presented here demonstrates that inhibition of activin A/TGFβ signalling can abrogate LMP1’s ability to enhance cell adhesion and to spread on fibronectin-coated slides (Fig. 8). Cells were cultured in the presence and absence of the type I activin A/TGFβ receptor inhibitor, SB431542, overnight prior to recovery and seeding as single cell suspensions onto fibronectin-coated slides for various time-points. Immunofluorescence staining for vinculin and active ERK-MAPK revealed an enhanced rate of focal adhesion formation in untreated LMP1-expressing cells compared to their untreated control counterparts. However, the addition of SB431542 retarded the rate of adhesion onto fibronectin in the LMP1-expressing cells, but not in their control counterparts (Fig. 8). These findings support the hypothesis that LMP1 is utilising activin A and/or TGFβ signalling to confer an invasive and migratory phenotype on epithelial cells by enhancing the rate of adhesion and spreading onto fibronectin, an ECM protein which is itself upregulated in response to activin A/TGFβ.

## Discussion

NPC is a highly metastatic disease in which LMP1 is believed to play an important role in the early stages of disease pathogenesis^4^,^6^. Findings presented here identify a novel mechanism by which LMP1 alters cellular signalling and behaviour *in vitro*, pointing to a mechanism by which it could contribute to tumour progression and the focal adhesion complex formation and turnover that comprises the early stages of metastasis in NPC *in vivo*. The ability of LMP1 to stimulate fibronectin expression and secretion (Fig. 1), and to enhance cellular adhesion and migration on fibronectin (Figs 6 and 7) is suggestive of a role for LMP1 in the metastatic process in NPC.

Previous studies have highlighted a role for LMP1 in abrogating TGFβ-mediated growth inhibition and Smad-dependent transcription through a mechanism involving NF-κB activation^30^,^31^. This current study demonstrates that LMP1 can enhance the expression and secretion of both activin A and TGFβ to induce fibronectin expression and secretion (Fig. 2), and that although LMP1 promotes Smad2 protein phosphorylation (Fig. 2), it inhibits Smad-dependent transcription (Fig. 3); moreover, the ability of LMP1 to induce fibronectin expression and secretion is Smad-independent (Fig. 3). Findings presented here provide a novel mechanistic insight into LMP1’s ability to exploit the well-documented “double-edged sword” nature of TGFβ signalling, whereby it attenuates TGFβ-mediated cytostasis, yet utilises the pro-fibrotic nature of this superfamily of cytokines by engaging an alternative signalling pathway via JNK/SAPK, in line with other published observations^33^. It is important to note that LMP1-mediated engagement of the JNK/SAPK pathway is itself independent of activin A/TGFβ signalling since inhibition using the type I receptor inhibitor, SB431542, failed to have any effect on intracellular levels of phosphorylated JNK (Fig. 4b).

Fibronectin is the major ligand for the α5β1 integrin receptor^45^ and is classically upregulated in response to keratinocyte wounding^46^. Increased levels of both α5 and active β1 integrins were observed on the cell surface of LMP1-expressing cells (Fig. 5a), along with the focal adhesion accessory protein, talin, which is only associated with activated integrins (Fig. 5b). These findings may suggest two things: 1) that LMP1 stimulates the production of a ligand to engage integrin signalling; and 2) that LMP1 increases the cell-surface expression of integrins to augment the outside-in signalling cascade activated by integrins. Further work is required to assess the extent of active integrin signalling in LMP1-expressing cells.

Previous observations have highlighted a role for ERK-MAPK signalling in LMP1’s ability to enhance cell motility and invasiveness^44^. Immunofluorescence staining for the focal adhesion protein, vinculin, highlights the increased rate of focal adhesion assembly and turnover in LMP1-expressing cells when compared with their control counterparts (Fig. 6). Moreover, an enhanced activation of ERK-MAPK, which is known to localise to points of focal adhesion^43^, is observed at points of focal adhesion in cells expressing LMP1 (Fig. 7). Allied to this, the requirement for activin A and/or TGFβ signalling in the adhesiveness of LMP1-expressing cells is highlighted, since treatment with the type I receptor inhibitor slows their rate of attachment and spreading onto fibronectin (Fig. 8). Since activin A and TGFβ are known to signal through the ERK-MAPK pathway, it would be convenient to hypothesise that this phenomenon merely represents an additional tier of signalling in this phenotypic effect of LMP1 expression. However, since inhibition of activin A and/or TGFβ fails to affect LMP1-mediated ERK activation (Fig. 8), these findings suggest that LMP1 can engage ERK-MAPK signalling without the requirement for a signal from activin A and/or TGFβ, and that it can utilise more than one signalling pathway to elicit its effects.

**Figure 9:**
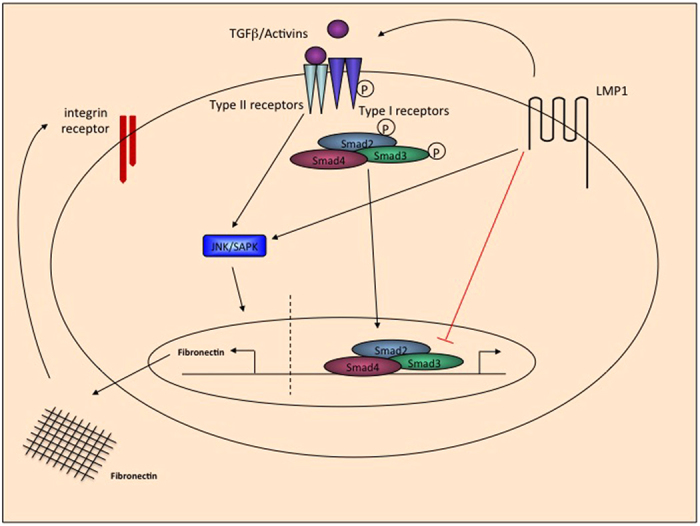
Schematic diagram summarising the mechanisms by which LMP1 induces fibronectin expression and deposition, and the downstream consequences of this phenomenon.

In summary, the findings presented here demonstrate that the EBV-encoded oncoprotein, LMP1, activates signalling through activin A and TGFβ1 to induce the expression, secretion and deposition of the extracellular matrix protein, fibronectin. Moreover, these effects are mediated via a Smad-independent mechanism involving JNK/SAPK, but LMP1-mediated JNK/SAPK itself is not abrogated by addition of the small molecule inhibitor for the activin A and TGFβ receptor, ALK5. In addition, LMP1 upregulates the cell surface expression and activation of integrins, resulting in enhanced adhesion to fibronectin. These interlinked observations are summarised in the schematic in Fig. 9. Taken together, these findings demonstrate the varied and far-reaching effects of LMP1-mediated activin A and TGFβ signalling in epithelial cells *in vitro*, pointing to a putative role for LMP1 in the metastatic potential of NPC cells *in vivo*. While further studies are required to examine this phenomenon in more detail, several studies have confirmed that EBV-positive NPC overexpress activin A and/or TGFβ^32^,^47^. This, coupled with the findings of Ma and colleagues^48^, who found a direct correlation between the levels of LMP1 and the expression of fibronectin, suggests that induction of fibronectin by activin A/TGFβ may contribute to tumour cell invasiveness *in vivo*.

Since fibronectin induction is a characteristic feature of wounded keratinocytes in tissue fibrosis, and fibrosis is itself a known precursor to neoplastic lesions, future work will look to utilise these mechanistic insights into the signalling intermediates required for such a response in order to identify putative therapeutic targets. Not only will this be of benefit for the treatment of EBV-associated cancers such as NPC, it will have far-reaching implications for the treatment of many other virus- and infectious agent-associated cancers, which bear many common features.

## Materials and Methods

### Cell lines and tissue culture

The human epithelial cell line SCC12F^49^ and a clone of SCC12F cells stably expressing LMP1 were cultured as described previously^50^. The HaCaT cell lines stably expressing the Tet repressor (TR) and the Tet-inducible Smad4 siRNA were a kind gift from Professor Caroline Hill, and were established as described previously^40^.

The recombinant human activin A protein (Peprotech, London, UK) was reconstituted in water to a concentration of 50 μg/ml, further diluted to a concentration of 10 μg/ml in PBS containing 2 mg/ml albumin, and used at a final concentration of 10 ng/ml. Recombinant human TGFβ1 (Peprotech) was reconstituted in 10 mM citric acid, pH 3.0, to a concentration of 50 μg/ml, further diluted to a concentration of 10 μg/ml in PBS containing 2 mg/ml albumin, and used at a final concentration of 10 ng/ml. The activin/TGFβ type I receptor inhibitor, SB431542 (Tocris, Bristol, UK), was dissolved in DMSO to a concentration of 10 mM and used at a final concentration of 10 μM. JNK/SAPK signalling was stimulated using anisomycin (Sigma Aldrich, Dorset, UK), reconstituted in DMSO to a concentration of 25 mg/ml and used at a final concentration of 250 ng/ml. The small molecule JNK/SAPK inhibitor, SP600125 (Sigma), was dissolved in DMSO at a concentration of 10 mM and used at a final concentration of 10 μM.

### Western blot analysis

Standard immunoblotting procedures^51^ were used to detect LMP1 (with mouse CS1-4 at 1:5), activin A (with mouse anti-inhibin βA at 1:50 [Serotec, Oxfordshire, UK]), β-actin (with mouse anti-β-actin at 1:20,000 [Sigma]), fibronectin (with mouse anti-fibronectin at 1:100 [Sigma]), phosphorylated Smad2 Ser465/Ser467 (with rabbit anti-pSmad2 at 1:250 [Cell Signaling Technology, Hitchin, UK]), Smad2 (with rabbit anti-Smad2 at 1:250 [Cell Signaling Technology]), Smad4 (with rabbit anti-Smad4 at 1:250 [Cell Signaling Technology]), phosphorylated JNK/SAPK (with rabbit anti-pJNK/SAPK at 1:500 [Cell Signaling Technology]), JNK/SAPK (with rabbit anti-JNK/SAPK at 1:500 [Cell Signaling Technology]).

### Quantitative PCR (qPCR)

RNA was extracted using EZ-RNA total RNA isolation kit (Geneflow, Lichfield, UK) and amplification by qPCR was performed using and ABI 7500 real-time PCR machine following standard procedures. A TaqMan primer probe set for fibronectin was purchased from Applied Biosystems (Hs01549937_m1), together with GAPDH as a standard control.

### ELISA

The presence of Activin A and TGFβ in the supernatants of cultured cells was determined by ELISA carried out following standard procedures, according to the manufacturers’ instructions (TGFβ – Promega, Southampton, UK; Activin A – R&D Systems, Abingdon, UK). Cells were cultured in serum-free medium supplemented with 0.5% BSA for 16 h prior to collection of conditioned medium.

### Transient transfections and luciferase reporter assays

Transient transfection was performed using the lipofectamine and plus reagent system according to manufacturer’s instructions (Life Technologies, Paisley, UK). The dual luciferase reporter assay (Promega) was carried out according to the manufacturer’s instructions, using the *Renilla* plasmid to control for transfection efficiency in addition to the following reporter constructs: pGL3 basic (Promega); ARE-Luc^38^; and CAGA-Luc^39^.

### Immunofluorescence staining

Cells were cultured in medium containing 0.5% BSA for 16 h prior to recovery as single-cell suspensions and maintained in suspension for 60 min in the presence and absence of the activin A/TGFβ inhibitor, SB431542. Cells (10^4^/100 μl) were plated onto slides coated with fibronectin (10 μg/ml) and allowed to adhere for 60 min. Slides were fixed in 4% paraformaldehyde, then permeabilised in 0.5% Triton X-100 (Sigma) prior to staining simultaneously with antiserum specific for vinculin (Sigma), talin (Abcam, Cambridge, UK) and active ERK-MAPK (Promega), followed by Alexa Fluor 488-conjugated anti-mouse and 534-conjugated anti-rabbit antibodies (Molecular Probes, Paisley, UK). Slides were mounted with DABCO and viewed using confocal laser microscopy.

### Flow cytometry to detect levels of cell surface integrins

Cells were cultured in serum-free medium supplemented with 0.5% BSA for 16 h prior to harvesting in a weak trypsin:EDTA solution (1:20) to avoid cleavage of cell surface receptors. 2 × 10^5^ cells per reaction were plated into a pre-chilled V-bottomed 96-well plate and centrifuged at 1500 × g for 3 min at 4 ^o^C. Cell pellets were washed twice in ice cold 1% FCS/PBS, then resuspended in 1% FCS/PBS containing a 1:50 dilution of active β1 integrin antibody and incubated on ice for 100 min in the dark. Cell pellets were washed three more times prior to resuspension in ice cold 1% FCS/PBS containing a 1:1000 dilution of the anti-mouse FITC-conjugated secondary antibody and incubated on ice for 1 hour in the dark. An anti-IgG antibody was included as an isotype control. Following three more washes, cell pellets were finally resuspended in 100 μl of ice cold 1% PFA/PBS and transferred to labelled 5 ml polystyrene round bottom Falcon tubes (BD Biosciences, Oxford, UK) where a further 400 μl of 1% PFA/PBS was added. Samples were read on a Coulter Epics XL-MCL^TM^ flow cytometer.

## Additional Information

**How to cite this article**: Morris, M. A. *et al*. The Epstein-Barr virus encoded LMP1 oncoprotein modulates cell adhesion via regulation of activin A/TGFβ and β1 integrin signalling. *Sci. Rep.* **6**, 19533; doi: 10.1038/srep19533 (2016).
